# Triple Therapy De-Escalation and Withdrawal of Inhaled Corticosteroids to Dual Bronchodilator Therapy in Patients with Chronic Obstructive Pulmonary Disease (COPD): A Systematic Review and Meta-Analysis

**DOI:** 10.3390/jcm13206199

**Published:** 2024-10-18

**Authors:** Edoardo Pirera, Domenico Di Raimondo, Antonino Tuttolomondo

**Affiliations:** Internal Medicine and Stroke Care Ward, Department of Promoting Health, Maternal-Infant, Excellence and Internal and Specialized Medicine (ProMISE) “G. D’Alessandro”, University of Palermo, 90133 Palermo, Italy; edoardo.pirera@unipa.it (E.P.); bruno.tuttolomondo@unipa.it (A.T.)

**Keywords:** chronic obstructive pulmonary disease (COPD), inhaled corticosteroids (ICSs), de-escalation therapy, acute exacerbation of COPD (AECOPD), pneumonia, eosinophils

## Abstract

**Background/Objectives:** The interpretation of evidence on the de-escalation of triple therapy with the withdrawal of inhaled corticosteroids (ICSs) to dual bronchodilator therapy with a long-acting muscarinic antagonist (LAMA) and a long-acting beta-agonist (LABA) in patients with chronic obstructive pulmonary disease (COPD) is conflicting. We evaluated the efficacy and safety of ICS discontinuation from LABA-LAMA-ICS triple therapy compared to its continuation. **Methods**: We searched PubMed, Embase, Scopus, Web Of Science, clinicaltrial.gov, and CENTRAL for RCTs and observational studies from inception to 22 March 2024, investigating the effect of triple therapy de-escalation with the withdrawal of ICSs to dual therapy on the risk of COPD exacerbation, pneumonia, and lung function. This study was registered with PROSPERO, CRD42024527942. **Results**: A total of 3335 studies was screened; 3 RCTs and 3 real-world non-interventional studies were identified as eligible. The analysis of the time to the first moderate or severe exacerbation showed a pooled HR of 0.96 (95% CI, 0.80–1.15; I^2^ = 77%) for ICS withdrawal compared to triple therapy continuation. The analysis according eosinophil levels showed that COPD subjects with ≥300 eosinophils/µL had a significant increase in the incidence of moderate or severe exacerbations when de-escalated to LABA/LAMA (pooled HR: 1.35, 95% CI: 1.00–1.82; I^2^: 56%). ICS withdrawal did not significantly affect the risk of mortality and pneumonia. **Conclusions:** The de-escalation of triple therapy with ICS withdrawal does not affect the main outcomes evaluated (moderate or severe exacerbations, change in trough FEV_1_). COPD patients with high blood eosinophils (≥2% or ≥300 cells/µL) are most likely to benefit from continuing triple therapy.

## 1. Introduction

Chronic obstructive pulmonary disease (COPD) is a progressive respiratory disease characterized by airflow limitation and persistent respiratory symptoms [1]. According to the most important consensus documents [1], the management of stable COPD mainly consists in the use of inhaled medications that have been shown to relieve symptoms, reduce exacerbations, and improve the quality of life [2]. One of the leading treatment strategies is the concept of triple therapy, which combines an inhaled corticosteroid (ICS), a long-acting muscarinic antagonist (LAMA), and a long-acting beta-agonist (LABA). Triple therapy is currently indicated for maintenance treatment in stable COPD patients experiencing frequent exacerbations and in patients with a significant symptomatic burden despite dual bronchodilator therapy [1,2]. Randomized clinical trials have shown that triple therapy can improve lung function and quality of life and reduce exacerbations compared to dual therapy [3,4,5,6].

Despite the widely accepted role of triple therapy, concerns have been raised about the safety of long-term ICS use, such as the potential risk of adverse events, particularly pneumonia. A systematic review and meta-analysis of three RCTs found a higher incidence of pneumonia in those who were randomized to triple therapy compared to the LABA/LAMA group (risk ratio: 1.53; 95% confidence interval (CI): 1.25–1.87; I^2^ = 19.7%) [7]. Conversely, another meta-analysis by Calzetta et al. [8] indicated that ICS discontinuation may result in an increased risk of severe exacerbations, impairment of lung function, and worsened quality of life, although the impact may not be clinically significant [8]. ICS add-on therapy to LABA_LAMA has been associated with lower mortality rates, particularly in patients with higher exacerbation rates and more severe clinical symptoms [1], but there are limited data on mortality after ICS withdrawal from triple therapy.

The current indication for ICS withdrawal from triple therapy to LABA-LAMA is limited to COPD patients with no history of exacerbations in the past year, and the recommendation had only moderate certainty of evidence [2]; the latest GOLD document [1] only states that it is appropriate to “review and assess, then adjust if necessary” after the initiation of therapy, without providing further guidance due to a lack of clear evidence. Many authors question the rationale for changing a therapy that provides significant patient benefit: there is a considerable lack of evidence on the effects of de-escalating from triple therapy to LABA/LAMA, and the available data paint an unclear picture. One of the main issues leading to the tapering of ICSs and the step-down to LABA/LAMA may be the need to balance the benefits of inhaled steroids against their potential risks, particularly pneumonia. Interestingly, evidence from “real-world” studies suggests that dual bronchodilator therapy is as effective as triple therapy for many patients, particularly those without a history of frequent exacerbations, but with a better safety profile [9,10], further increasing the need for reliable data on the safety and effectiveness of a de-escalation approach in selected patients, testing the hypothesis that, in certain groups of patients, ICSs can be withdrawn from triple therapy without increasing the risk of exacerbation or death.

For the purpose of this meta-analysis, we searched for relevant randomized controlled clinical trials and observational studies that evaluated the efficacy and safety of ICS withdrawal from triple therapy (ICS/LAMA/LABA) by measuring moderate and severe exacerbations, lung function, and adverse events, including pneumonia and all-cause mortality. We also evaluated the effects of the de-escalation strategy in specific subgroups of COPD patients, namely according to different blood eosinophil counts.

## 2. Materials and Methods

### 2.1. Search Strategy and Selection Criteria

The inclusion criteria for this systematic review with meta-analysis were limited to studies that met all of the following eligibility criteria: (1) RCTs or observational studies that enrolled adults aged ≥ 40 years with a diagnosis of COPD; (2) those comparing ICS withdrawal from triple therapy with LABA/LAMA regimen versus the continuation of LABA/LAMA/ICS; (3) follow-up period of at least of six months; and (4) those reporting any of the outcomes of interest. We excluded studies that met at least one of the following exclusion criteria: (1) no control group; (2) overlapping study population. We systematically searched MEDLINE (via PubMed), Embase, Scopus, Web Of Science, and Cochrane Central Register of Controlled Trials (CENTRAL) for eligible studies from inception to 22 March 2024. The search term strategy included a combination of synonyms of “COPD”, “glucocorticoid”, “inhaled”, and “withdrawal”. The complete electronic search strategy is reported in Supplementary S1. Only publications in English were considered. E.P. and D.D.R. independently reviewed each title and abstract for potential eligibility. Disagreements were resolved by consensus among the three authors. Finally, we report the results of the search strategy according to the Preferred Reporting Items for Systematic Reviews and Meta-Analysis (PRISMA) statement guidelines [11].

For the purpose of this meta-analysis, we extracted and evaluated data for the following: (1) moderate or severe COPD exacerbation; (2) change from baseline in forced respiratory volume in the first second (FEV_1_) recorded anytime and the “trough FEV_1_” (i.e., the FEV_1_ measurement before the next inhalation of study drug and ~24 h after the last inhalation was evaluated); (3) incidence of pneumonia; (4) incidence of all-cause mortality. A detailed definition of the outcomes of interest is provided in Supplementary S2. Additionally, we evaluated the effects of the de-escalation strategy compared to the continuation of LABA/LAMA/ICS in specific subgroups of COPD patients. Specifically, we performed subgroup analyses according to different blood eosinophil thresholds: (1) <2%; (2) ≥2%; (3) <150 cells/µL; (4) ≥150 cells/µL and <300 cells/µL; (5) ≥300 cells/µL.

### 2.2. Data Analysis

E.P. and D.D.R. independently extracted baseline data for the studies included in the meta-analysis. Summary estimates for binary outcomes were reported as pooled hazard ratio (HR) or rate ratio (RR) with 95% of confidence intervals for time-to-event and event rate data, respectively. For pooled continuous data, we reported the treatment effect as the mean difference. Dichotomous outcomes were reported as risk ratios with 95% confidence intervals. All analyses were performed using the random effects model and heterogeneity was tested using the Cochran Q test and I^2^ statistics. RevMan Web (Nordic Cochrane center, The Cochrane collaboration, Copenhagen, Denmark) was used for statistical analysis. The quality assessment of RCT was evaluated using “The Cochrane tool for assessing risk of bias in randomised trials” (RoB2) [12] and, for non-randomized trials, we used the “Risk Of Bias In Non-randomised Studies-of Interventions” (ROBINS-I) [13]. To evaluate the robustness and conclusiveness of the findings, sensitivity analyses using the jackknife method were conducted using R 4.4.0 (Foundation for Statistical Computing, Vienna, Austria). This involved systematically excluding individual studies and recalculating the meta-analysis using the remaining pooled estimates. By iteratively omitting each study and re-evaluating the pooled estimates, we aimed to determine if any single study disproportionately influenced the overall results [14]. Finally, a subset analysis was performed by restricting the analysis to RCTs. This approach aimed to mitigate potential biases introduced by including non-RCTs in the primary analysis. This systematic review and meta-analysis was registered on PROSPERO, CRD42024527942. There was no funding source for this study.

## 3. Results

**Figure 1:**
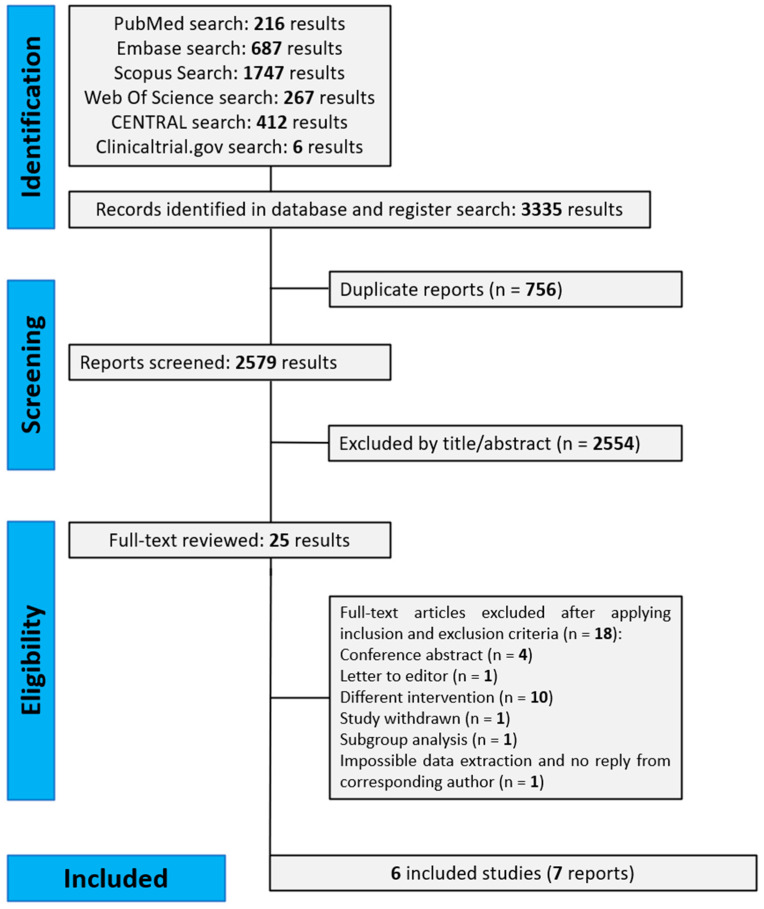
PRISMA flow diagram of the systematic literature search, study selection, and reasons for exclusion.

Our literature search initially yielded 3335 results. We subsequently excluded 3310 records after removing duplicates (n: 756) and after the title/abstract inspection (n: 2554). A total of 25 articles was fully reviewed (Supplementary S3). Ultimately, the results were extracted from three RCTs [15,16,17,18] and three “real-world” non-interventional studies [19,20,21]. Two separate reports from the “Withdrawal of Inhaled Steroids during Optimized Bronchodilator Management” (WISDOM) trial were included in our meta-analysis: Magnussen et al. [15] reporting on the full study population and Watz et al. [16] presenting a post hoc subgroup analysis based on eosinophil count. Figure 1 shows the PRISMA flow diagram of the systematic literature search, study selection, and reasons for exclusion.

### 3.1. Characteristics of the Studies Included in This Meta-Analysis

Table 1 lists the main characteristics of the studies included in this meta-analysis. All participants were at least 40 years of age (mean age from 63.8 to 70.8 years) and were current or former smokers. In four studies [15,16,17,18,19], a history of exacerbations in the previous year was required. Given the non-interventional design of the three “real-world” studies [19,20,21], participants continued triple therapy or de-escalated to a LABA/LAMA depending on physician judgment or randomly allocated in the three RCTs [15,16,17,18]. Finally, one study [19] included three different COPD cohorts in the analysis: (1) Cohort 1 that specifically met the inclusion and exclusion criteria of the WISDOM trial [15]; (2) a “real-world” COPD cohort aged 35 years or older (Cohort 2); (3) Cohort 3 consisting of COPD patients aged 35 years or older that met at least one exclusion criteria of the WISDOM trial [15]. The full inclusion and exclusion criteria of WISDOM are reported in the original article [15]. According to our pre-defined inclusion criteria, only Cohort 1 was eligible for inclusion in our meta-analysis. All participants received a COPD diagnosis confirmed by spirometry and a post-bronchodilator ratio of FEV_1_ and FVC < 0.7.

### 3.2. Pooled Analysis of Time to the First Moderate or Severe Exacerbation and Event Rate for ICS Withdrawal Compared with Continuation of Triple Therapy

**Figure 2:**
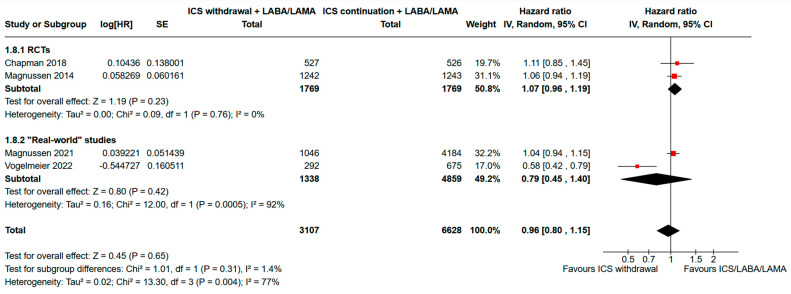
Analysis of the time to the first moderate or severe exacerbation for ICS withdrawal compared to triple therapy continuation. ICS: inhaled corticosteroid; LABA: long-acting beta-agonist; LAMA: long-acting muscarinic antagonist; HR: hazard ratio; RCTs: randomized controlled trials; SE: standard error; 95% CI: 95% confidence interval [15,18,20,21].

The pooled analysis of time-to-event data for “moderate or severe acute exacerbations” that included 9735 participants is showed in Figure 2. The analysis of time to the first moderate or severe exacerbation showed a pooled HR of 0.96 (95% CI, 0.80-1.15; I^2^ = 77%) for ICS withdrawal compared to the continuation of triple therapy. The subset analysis including only RCTs showed a pooled HR of 1.07 (95% CI, 0.96–1.19) with low heterogeneity (I^2^ = 0%).

**Figure 3:**
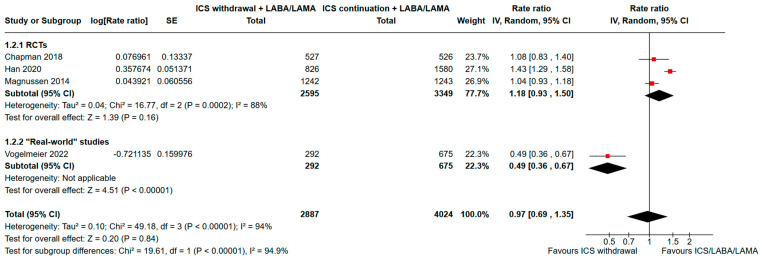
Forest plot of meta-analysis comparing event rate for severe acute exacerbations in de-escalation from triple therapy with ICS withdrawal vs. triple therapy continuation. ICS: inhaled corticosteroid; LABA: long-acting beta-agonist; LAMA: long-acting muscarinic antagonist; RCTs: randomized controlled trials; SE: standard error; 95% CI: 95% confidence interval [15,17,18,20].

A further analysis of the event rate in 6911 patients yielded an RR of 0.97 with substantial heterogeneity (95% CI, 0.69-1.35; I^2^ = 94%; see Figure 3). Furthermore, the subset analysis of RCTs showed a consistent result with a pooled RR of 1.18 (95% CI, 0.93–1.50) with substantial heterogeneity (I^2^ = 88%).

**Figure 4:**
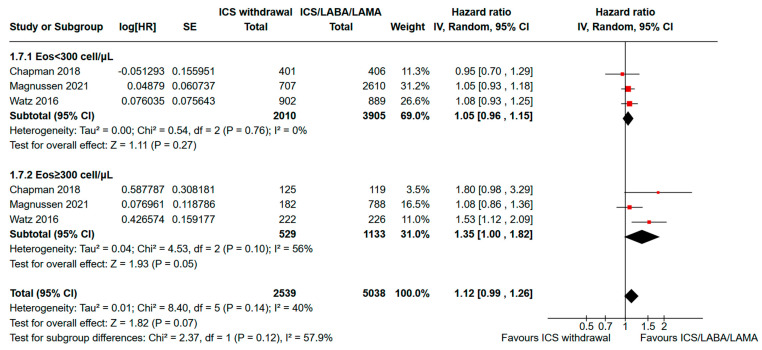
Analysis of the time to the first moderate or severe exacerbation for ICS withdrawal compared to triple therapy continuation for COPD subjects with ≥300 eosinophils/µL vs. <300 eosinophils/µL. ICS: inhaled corticosteroid; LABA: long-acting beta-agonist; LAMA: long-acting muscarinic antagonist; HR: hazard ratio; SE: standard error; 95% CI: 95% confidence interval [16,18,21].

The subgroup analysis according to the eosinophil count and including 7577 participants (2539 without ICSs and 5038 in triple therapy) from two RCTs [16,18] and one observational study [21] showed a significant increase in the risk of moderate or severe exacerbations among COPD patients with a blood eosinophil counts ≥300 cells/µL who underwent de-escalation from triple therapy to LABA/LAMA (Figure 4). The pooled HR was 1.35 (95% CI: 1.00–1.82), with an I^2^ of 56% indicating moderate heterogeneity. Conversely, the same intervention did not yield a statistically significant difference in exacerbation rates among COPD patients with eosinophil counts <300 cells/μL (pooled HR: 1.05, 95% CI 0.96–1.15; I^2^: 0%). The *p*-value interaction was not statistically significant (*p* = 0.12).

The subset analysis of the RCTs further confirmed these results. For COPD patients with eosinophils <300 cells/μL, the pooled HR was 1.05 (95% CI, 0.92–1.20), with no statistically significant difference between ICS withdrawal and ICS/LABA/LAMA (*p* = 0.45) and low heterogeneity (I^2^ = 0%). Conversely, for COPD patients with eosinophils ≥300 cells/μL, the pooled HR was 1.58 (95% CI, 1.20–2.09) with low heterogeneity (I^2^ = 0%), indicating a statistically significant increased risk of moderate or severe exacerbations after ICS withdrawal. Significant subgroup differences were observed (*p* = 0.009), with high heterogeneity (I^2^ = 85.3%), suggesting that COPD patients with an absolute eosinophil count ≥300 cell/μL may have a greater clinical benefit continuing ICS/LABA/LAMA rather than de-escalating to LABA/LAMA. Data are available in Supplementary S4.1.

For the analysis of event rates according to circulating eosinophils, we extracted available data from the RCTs of Chapman et al. and Watz et al. [16,18].

**Figure 5:**
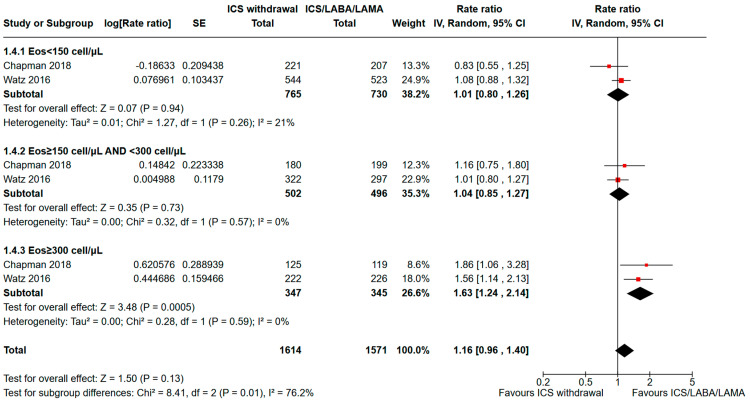
Analysis of moderate or severe exacerbation rates for ICS withdrawal compared to triple therapy continuation for COPD subjects with < 150 eosinophils/µL vs. ≥300 eosinophils/µL vs. intermediate eosinophil levels (≥150 and <300/µL). ICS: inhaled corticosteroid; LABA: long-acting beta-agonist; LAMA: long-acting muscarinic antagonist; SE: standard error; 95% CI: 95% confidence interval [16,18].

In COPD patients with low circulating eosinophils, the same intervention did not show a significant difference in exacerbation rates, as shown in Figure 5: For COPD patients with eosinophils <150 cell/μL or ≥150 cell/μL and <300 cell/μL, the pooled RR were 1.01 (95% CI: 0.80–1.26; I^2^ = 21%) and 1.04 (95% CI: 0.85–1.27; I^2^ = 0%), respectively. For COPD patients with absolute eosinophils ≥300 cells/µL, the pooled RR was 1.63 (95% CI: 1.24–2.14; I^2^: 0%). The test for subgroup differences between the three absolute eosinophil count thresholds suggests that there is a statistically significant subgroup effect (*p* interaction = 0.01, I^2^: 76.2%).

The same results emerged from the subgroup analysis according to the percentage of eosinophil count (<2% vs. ≥2%), including available data from RCTs of Chapman et al. and Watz et al. [16,18]. Data are available in Supplementary S4.2.

### 3.3. Pooled Analysis of FEV_1_ Variation

Data from two RCTs [15,18] were extracted for the pooled analysis of trough FEV_1_ variation between the two therapeutic strategies. The analysis showed a statistically significant difference toward the ICS/LABA/LAMA group with a mean difference of 34.7 mL (95% CI: 15.84 to 53.57 mL) and low heterogeneity (I^2^: 0%).

**Figure 6:**
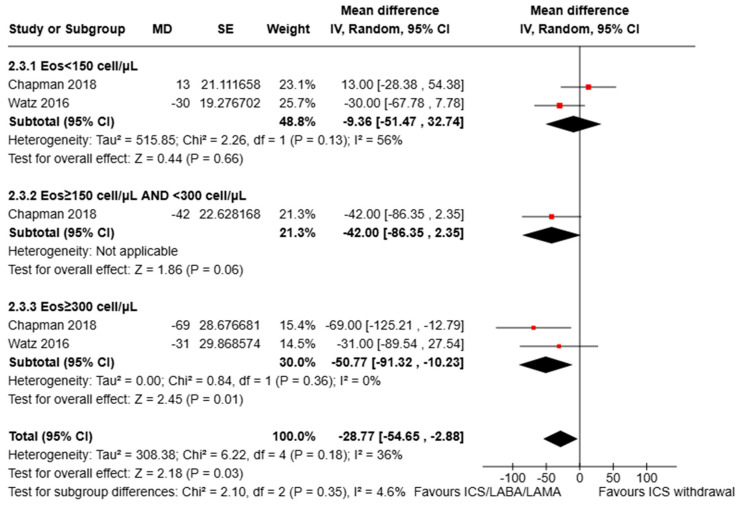
Analysis of the trough FEV_1_ variation for ICS withdrawal compared to triple therapy continuation for COPD subjects with <150 eosinophils/µL vs. ≥300 eosinophils/µL vs. intermediate eosinophil levels (≥150 and <300/µL). ICS: inhaled corticosteroid; LABA: long-acting beta-agonist; LAMA: long-acting muscarinic antagonist; MD: mean difference; SE: standard error [16,18].

Subgroup analysis according to eosinophil count showed no statistically significant decrease in trough FEV_1_ for COPD patients with circulating eosinophils (<150 cell/μL: mean difference: −9.36, 95% CI: −51.47 to 32.74; I^2^ = 56%; ≥150 cell/μL and <300 cell/μL: mean difference: −42.00, 95% CI: −86.35 to 2.35), but a significant reduction for those with a circulating eosinophil count ≥300 cells/µL (mean difference: −50.77 mL, 95% CI: −91.32 to −10.23; I^2^ = 0%) (data displayed in Figure 6). The test for subgroup differences indicated that there was no statistically significant subgroup effect (*p* interaction = 0.35).

Similar results were found according to the percentage of eosinophil count (<2% vs. >2%). Data are available in Supplementary S4.3.

For the meta-analysis of change from baseline in FEV_1_ recorded anytime, data from two observational studies were extracted [19,21]. No significant differences were observed between those who continued triple therapy and those who descaled to LABA/LAMA. Full data are reported in Supplementary S4.4.

### 3.4. Evaluation of Safety Outcomes: Risk of Pneumonia and All-Cause Mortality

**Figure 7:**
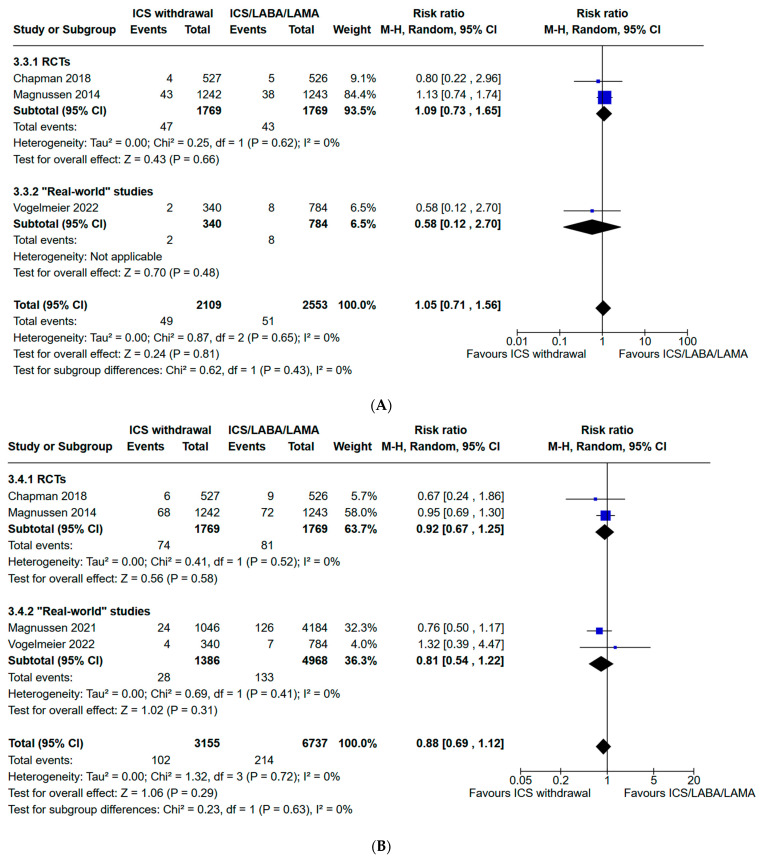
(**A**). Forest plot of meta-analysis for all-cause mortality in de-escalation from triple therapy with ICS withdrawal group vs. triple therapy continuation group. (**B**). Forest plot of meta-analysis for the risk of pneumonia in de-escalation from triple therapy with ICS withdrawal group vs. triple therapy continuation group. ICS: inhaled corticosteroid; LABA: long-acting beta-agonist; LAMA: long-acting muscarinic antagonist; RCTs: randomized controlled trials; 95% CI: 95% confidence interval [15,18,20,21].

For the safety outcomes, we included three studies [15,18,20] for “all-cause mortality” and four studies [15,18,20,21] for the risk of pneumonia with 4662 and 9892 participants, respectively. “All-cause mortality” occurred in 49 patients in the ICS withdrawal group (2.3%) and in 51 patients in the ICS/LABA/LAMA group (2%). Compared to ICS withdrawal, ICS/LABA/LAMA showed no increase in the risk of “all-cause mortality” (pooled risk ratio: 1.05, 95% CI: 0.71–1.56; I^2^: 0%; Figure 7A) with low heterogeneity among studies (I^2^: 0%). Pneumonia occurred in 102 patients in the ICS withdrawal group (3.2%) and in 214 patients in the ICS/LABA/LAMA group (3.1%). Withdrawal of ICSs in favor of LABA/LAMA did not significantly affect the risk pneumonia (pooled risk ratio: 0.88, 95% CI: 0.69–1.12; I^2^: 0%; Figure 7B). The subset analysis with RCTs showed consistent findings compared to the primary analysis.

### 3.5. Sensitivity Analyses and Risk of Bias Assessment

The jackknife sensitivity analysis showed that one study excessively affected the pooled estimates by introducing heterogeneity into the meta-analysis. Specifically, by omitting the “Outpatient care with long-acting bronchodilators: COPD register in Germany” (DACCORD) study [20], we observed a trend toward the reduction in exacerbation rates in COPD patients who discontinued ICSs compared to those who continued triple therapy, although this did not reach statistical significance (pooled HR: 1.05, 95% CI: 0.97–1.14; I^2^: 0% vs. I^2^: 77%; pooled RR: 1.18, 95% CI: 0.93–1.50; I^2^: 88% vs. I^2^: 94%). Finally, the pooled estimate remained consistent for all-cause mortality and pneumonia rates when the meta-analysis was repeated by omitting one study at a time, indicating the robustness of our results (see full analysis in Supplementary S5).

Regarding the assessment of risk of bias, two RCTs [17,18] were deemed at low risk of bias and one RCT [15] with some concerns according the RoB2 tool. For observational studies, the overall risk of bias judgement was deemed at serious risk of bias according to ROBIN-I (see Supplementary S6).

## 4. Discussion

In the present meta-analysis, we systematically evaluated the efficacy and safety of de-escalation with ICS withdrawal from ICS/LABA/LAMA to LABA/LAMA compared to the continuation of triple therapy. The main findings of our meta-analysis are that there was no significant increase in moderate or severe exacerbations as a result of ICS withdrawal, nor was there a change in trough FEV_1_ from baseline. Moreover, no significant differences were observed in our safety outcome analysis, including data on all-cause mortality and pneumonia risk. In our meta-analysis, we first evaluated specific subgroups of patients according to different eosinophil thresholds. We found that COPD patients with ≥300 eosinophil/µL or greater than 2% experienced a significant increase in exacerbations and a greater decline in lung function when de-escalating from ICS/LABA/LAMA to LABA/LAMA, suggesting that ICS withdrawal should be managed with greater attention in this subgroup considering the continuation of triple therapy.

We found only one meta-analysis similar to this one in a systematic review of the literature, dated 2021 by Koarai et al. [22]. However, this meta-analysis is significantly different from ours; in fact, Koarai et al. evaluated the efficacy and safety of ICS/LAMA/LABA versus LAMA/LABA therapy but considering both ICS add-on to LAMA/LABA and ICS withdrawal from triple therapy. Koarai et al. [22] included only two trials that evaluated the effects of ICS withdrawal from ICS/LAMA/LABA. Compared to the ICS add-on protocol, there were no differences between ICS/LAMA/LABA and LAMA/LABA in the rate of exacerbations and total and serious adverse events, including pneumonia and mortality. These results are consistent with ours. However, no subgroup analysis based on eosinophils was performed in that paper. So, our paper extends the quantitative analysis performed in this previous meta-analysis [22] and is, to our knowledge, the first comprehensive analysis attempting to summarize the existing evidence on the de-escalation strategy from ICS/LABA/LAMA to LABA/LAMA in the treatment of stable COPD, including a subgroup analysis according to the eosinophil count. Furthermore, while a previous network meta-analysis of four RCTs and 21,809 patients [23] showed that ICS/LABA/LAMA significantly reduced the risk of moderate or severe exacerbations compared to dual therapy (ICS/LABA or LAMA/LABA), a comprehensive synthesis of findings from RCTs and real-world studies of “ICS withdrawal” studies investigating de-escalation from ICS/LABA/LAMA to LABA/LAMA was still absent.

Despite the importance of the clinical problem addressed in this meta-analysis and the very large number of patients potentially involved, our literature search found a limited number of studies and a significant methodological heterogeneity among them, encompassing variations in study design, patient populations, and ICS withdrawal protocols. Despite its clinical significance [24], we found, across the included studies, a great heterogeneity on the percentage of current smokers included. There was a notable degree of heterogeneity in the duration of triple therapy prior to enrolment in the trials considered: at least three months in four trials [17,18,19,20] and six weeks in the WISDOM trial [15,16], although one real-world trial [21] included patients regardless of their duration of triple therapy. In addition, the studies considered as “triple therapy” both when ICSs, LABA, and LAMA were administered in a single inhaler and in separate inhalers; nevertheless, this issue has been reported by several authors as a potential determinant of differential benefits for subjects [25,26].

As enlightened by our jackknife sensitivity analysis, such notable variance in pooled estimates was predominantly attributable to the inclusion of one study characterized by distinct methodological approaches. Regarding the analysis of the exacerbation rates, exclusion from the meta-analysis of the DACCORD trial [20] yielded a substantial change in the pooled estimates. The DACCORD trial was a longitudinal, non-interventional “real-world” study conducted in three different cohorts [20,27]. Only cohort 3 met our inclusion criteria (received triple therapy for ≥6 months), and then each patient’s physician decided to continue triple therapy or switch to a LABA/LAMA; so, the data were included in this meta-analysis and discussed in this paper. Briefly, patients de-escalated to LABA/LAMA had a lower incidence of both moderate and severe exacerbations compared to those in the ICS/LABA/LAMA group (HR: 0.58, 95% CI: 0.42–0.79), improved in symptoms (absolute change from baseline in CAT score −2.0 for LABA/LAMA group and −1.0 for ICS/LABA/LAMA group, *p* = 0.003) and had a lower incidence of adverse events. These results from the DACCORD trial [20] differ significantly from the other available evidence and need to be interpreted with caution. First, the “non-interventional” design could significantly contribute to the observed results. Second, the observed differences may be due to the severity of COPD in the patient population treated with ICS/LABA/LAMA. In fact, the mean predicted percentage of FEV_1_ (57.7%) was lower in the triple therapy group than in the LABA/LAMA group (66.9%), indicative of more severe disease at baseline. This severity is further highlighted by the higher frequency of exacerbations in the 12 months prior to study entry in the ICS/LABA/LAMA group (48.9%) than in the LABA/LAMA cohort (43.5%). Despite the methodological limitations of the non-interventional design, the DACCORD trial probably more closely reflects the “real-world” clinical management of patients with COPD on long-term triple therapy and demonstrates the clinical propensity of physicians to continue triple therapy for patients with unstable disease. This clinical decision-making is logically consistent with a cautious approach to managing patients who are more prone to exacerbations. Consequently, non-frequently exacerbating COPD patients were considered suitable candidates for ICS withdrawal and clinicians were dissuaded from de-escalating treatment in patients with a history of frequent exacerbations prioritizing disease control and prevention of further exacerbations. Of note, there is no mention of whether eosinophil counts had a role in de-escalating to LABA/LAMA or remaining on triple therapy.

Also, in consideration of the extensive debate over the past years regarding the role of ICSs in COPD [28,29,30,31,32], the analysis of data elaborated in the present meta-analysis suggests several considerations of potential interest for clinicians.

(1) The actual relevance of using trough FEV_1_ as an outcome measure in COPD patients may be questionable. Data from different studies appear to be influenced by the methodology used and, most importantly, no variation reaches the 100 mL target, which is the well-recognized “minimal clinically important difference” (MCID) [33,34]. Therefore, although the assessment of this measure could be of relevance to demonstrate the therapeutic effect, it does not necessarily translate for the patients in a clinical improvement, not leading to a significant improvement in perceived quality of life.

(2) In accordance with established protocols, five of the six studies included in our meta-analysis excluded only patients with a “current” diagnosis of asthma, whereas in the “Study to Understand the Safety and Efficacy of ICS Withdrawal from Triple Therapy in COPD” (SUNSET) study [18] subjects with a history of asthma were excluded a priori (see Table 1). The lack of a precise definition of asthma history can result in a wide range of patient profiles, from well-controlled patients with asthma to those with active asthma symptoms. Thus, the difference between protocols that exclude subjects with “current asthma” or with “history of asthma” raises methodological considerations because of the different role covered by ICS therapy in COPD subjects versus those with asthma-COPD overlap (ACO) [35]. Although ICS withdrawal may be feasible in certain asthma phenotypes [36], the possible inclusion of patients with ACO in studies primarily targeting COPD can significantly affect the generalizability of the findings, leading to an overestimation of the benefits of ICS therapy or an underestimation of the risks associated with discontinuation.

(3) Our findings provide a small but perhaps significant addition to the debate regarding the long-term safety of ICSs in COPD subjects. Previous meta-analyses have found a strong association between ICS use and the risk of pneumonia [7,37]. With the limitations imposed by the paucity of available studies, we found no safety concern for triple therapy versus LABA-LAMA de-escalation. This may be an important issue that needs to be further addressed in the future, especially in more targeted populations, including those with varying disease severity and exacerbation patterns. Our divergent results with the existing literature denote an urgent need for well-powered trials to identify COPD patients in whom the advantages of ICSs substantially outweigh the risks.

(4) The clinical significance of blood eosinophil levels in COPD is a subject of ongoing debate. Since the 2019 GOLD report [38] to the latest one [1], the peripheral blood eosinophil count has been stated as a parameter for guiding or modifying COPD treatment, including the use of ICS-based regimens. As observed in RCTs, in COPD patients not receiving ICS-containing regimens, the likelihood of exacerbation increases with the increase in eosinophil blood counts [39,40]. Conversely, in the FLAME study, the correlation between ICS therapeutic response and blood eosinophil level was not significant in COPD patients when comparing ICS/LABA to LABA/LAMA regimens in terms of exacerbation reduction. The LABA/LAMA combination was as effective as ICS/LABA in reducing exacerbation rates, regardless of the baseline eosinophil count (<2% or ≥2%) [41,42]. This is in contrast to the results of our quantitative analysis aggregating the results of the WISDOM and SUNSET trials [16,18], which form the basis of the most recent recommendations. For example, the European Respiratory Society (ERS) indicates a conditional recommendation for the withdrawal of ICSs in patients with COPD without a history of frequent exacerbations and a strong recommendation not to withdraw ICSs in patients who have a blood eosinophil count ≥300 eosinophils/μL, irrespective of the history of exacerbations [43]. Analogously, the American Thoracic Society’s guidelines do not advocate for or against the use of inhaled corticosteroids as an add-on therapy to long-acting bronchodilators for COPD patients with blood eosinophilia, except for those with a history of exacerbations [2]. The absence of a definitive recommendation underscores the need for further research in this area.

This study has several limitations. First, there is significant methodological heterogeneity among the included studies, such as protocols of ICS withdrawal (abrupt or stepwise) and the inclusion of subjects with a history of asthma and history of exacerbations in the previous 12 months. Second, the non-interventional design of the real-world studies included introduces potential biases that could influence outcomes, such as physician judgment in continuing or withdrawing ICSs. Third, the varying duration of triple therapy prior to study enrollment and differences in the administration of ICSs, LABA, and LAMA—whether in single or separate inhalers—could contribute to differential benefits observed across studies. Fourth, the varying duration of follow-up ranging from six and 12 months may have been inadequate to observe the outcomes. Fifth, due to the limited data available in the literature, it was not possible to perform a subgroup analysis based on the history of exacerbations that could be of great clinical relevance. Finally, due to the paucity of the included studies (<10 studies), we were not able to perform meta-regression and Egger’s test or funnel plots, which explore potential sources of heterogeneity and publication bias, respectively. This was conducted in accordance with the “Cochrane Handbook for Systematic Reviews of Interventions” (Sections 10.11.4 and 10.10.4.3) [44]. However, as highlighted by our meta-analysis, these limitations deserve to be appropriately explored with further prospective trials to facilitate more reliable comparisons and robust conclusions. Finally, we must acknowledge that eosinophil-directed therapy may not be a suitable option for all patients. It is important to note that COPD phenotypes are highly heterogeneous and that individualized therapy, taking into account several treatable traits, is imperative. It should also be noted that peripheral blood eosinophil count is a relatively variable parameter with moderate reproducibility. We evaluated both the absolute count (>300 cells) and the percentage count (>2%), but this limitation needs to be addressed.

## 5. Conclusions

A small body of evidence suggests that the de-escalation of triple therapy with ICS withdrawal to dual bronchodilator therapy in patients with COPD does not affect the main outcomes (moderate or severe exacerbations, change from baseline in trough FEV_1_). COPD patients with high blood eosinophils, namely ≥2% and ≥300 cells/µL, are most likely to benefit from continuing triple therapy and should not de-escalate to LABA/LAMA, as we found a greater risk of exacerbation and FEV_1_ decline, although the magnitude of change was far from the MCID threshold of 100 mL. In terms of adverse outcomes, including mortality and the risk of pneumonia, these events do not appear to be affected by ICS withdrawal. This suggests that there is no evidence that long-term use of ICSs is unsafe in this category of patients. The results of this meta-analysis support the current approach of consensus documents and ongoing clinical recommendations [1,2,41], which advocate the use of eosinophil counts to guide COPD maintenance therapy to provide a more precise and individualized therapeutic approach, including de-escalation treatments for patients with lower exacerbation risk profiles. Further research toward tailoring the inhaler strategy according to patient characteristics is needed.

## Figures and Tables

**Table 1:** Baseline characteristics of included studies.

1st Author, Year, Ref	Identifier	Design	Total Population (n)	Intervention	Control Group	Patient Characteristics	History of Exacerbation	Asthma	Age (Mean ± SD)	Male (%)	Current Smoker (%)	Post-Bronchodilator FEV1 (% Predicted)	Study Duration (Weeks)
Magnussen, 2014 Watz, 2016 [15,16]	WISDOM, NCT00975195	RCT	2485	Tiotropium 18 µg ODSalmeterol 50 µg BIDFormoterol 500 µg BID(Separate inhalers)	Tiotropium 18 µg OD Salmeterol 50 µg BID(Separate inhalers)	40 years old with FEV_1_ < 50% and FVC < 70%	≥1 AE in the 12 months before screening	History: not reported; Current: Excluded	63.8 ± 8.5	82.5	66.6	34.2 ± 11.0	52 weeks
Han, 2020 [17]	IMPACT, NCT02164513	RCT	2406	Fluticasone furoate 100 µgUmeclidinium 62.5 µgVilanterol 25 µg OD(Fixed inhaler)	Umeclidinium 62.5 µgVilanterol 25 µg OD(Fixed inhaler)	40 years old with FEV_1_ < 50% and ≥1 moderate or severe AE or 50% ≤ FEV_1_ < 80% and ≥ 2 moderate or ≥ 1 severe AE	≥1 AE in the 12 months before screening	History: Included; Current: Excluded	N/A	N/A	N/A	N/A	52 weeks
Chapman, 2018 [18]	SUNSET, NCT02603393	RCT	1053	Fluticasone furoate 500 µg BIDTiotropium 18 µg ODSalmeterol 50 µg BID(Separate inhalers	Glycopyrronium 50 µg ODIndacaterol 110 µg BID(Separate inhalers)	40 years old with moderate to severe COPD	≤1 moderate or Severe AE in the previous year	History: Excluded; Current: Excluded	65.3 ± 7.80	70.6	N/A	56.6 ± 9.97	24 weeks
Magnussen, 2021 [21]	EUPAS30851	Non-RCT	5230	ICS/LABA/LAMA	LABA/LAMA	40 years old with COPD	N/A	History: Included; Current: Included	70.8 ± 9.9	55.4	34.7	54.8 ± 22.2	52 weeks
Vogelmaier, 2022 [20]	DACCORD EUPAS4207	Non-RCT	1124	ICS/LABA/LAMA	LABA/LAMA	40 years old with COPD	N/A	History: Excluded; Current: Excluded	69.13 ± 9.42	57.7	31.13	60.5 ± 23.7	52 weeks
Whittaker, 2022 [19]	N/A	Non-RCT	6008	ICS/LABA/LAMA	LABA/LAMA	Same as WISDOM	Same as WISDOM	History: not reported; Current: Excluded	67.9 ± 9.1	61.2	50.5	N/A	At least six months

N/A: not available, applicable, or stated; ICS: inhaled corticosteroid; LAMA: long-acting muscarinic antagonist; LABA: long-acting beta-agonist; OD: omni die; BID: bis in die; FEV_1_: forced expiratory volume in the 1st second; FVC: forced vital capacity; AE: acute exacerbation; RCT: randomized controlled trial; SD: standard deviation; Based on the available data, only RCTs could be traced back to the actual inhalation therapy administered. For non-RCTs, this information is not available.

## Data Availability

The data that support the findings of this study are available from the corresponding author, upon reasonable request.

## Supplementary Materials

The following supporting information can be downloaded at: https://www.mdpi.com/article/10.3390/jcm13206199/s1. Supplementary S1: Search and study selection strategy. Supplementary S2: Definition of the outcomes of interest in the studies included in this meta-analysis [45]. Supplementary S3: List of studies excluded at the full-text screening stage with the reason for exclusion. Supplementary S4: Forest plot of additional data from analyzed outcomes. Supplementary S5: Jackknife sensitivity analyses. Supplementary S6: Risk of bias assessment.
