# Supplementary material for: Triple Therapy De-Escalation and Withdrawal of Inhaled Corticosteroids to Dual Bronchodilator Therapy in Patients with Chronic Obstructive Pulmonary Disease (COPD): A Systematic Review and Meta-Analysis
Source: J Clin Med. 2024 Oct 18;13(20):6199. doi: 10.3390/jcm13206199 (PMC11508213; doi:10.3390/jcm13206199)
Supplement: Supplementary file 1 [file jcm-13-06199-s001.zip › jcm-3214726-supplementary.pdf]

Supplementary S1. Search and study selection strategy.

Full search term for Embase, then translated for the other database: from inception to March 2024.

#1 ('chronic airflow obstruction' OR 'chronic airway obstruction' OR 'chronic obstructive bronchopulmonary disease' OR 'chronic obstructive lung disorder' OR 'chronic obstructive pulmonary disease' OR 'chronic obstructive pulmonary disorder' OR 'chronic obstructive respiratory disease' OR 'chronic pulmonary obstructive disease' OR 'chronic pulmonary obstructive disorder' OR 'copd' OR 'lung chronic obstructive disease' OR 'lung disease, chronic obstructive' OR 'obstructive chronic lung disease' OR 'obstructive chronic pulmonary disease' OR 'obstructive lung disease, chronic' OR 'pulmonary disease, chronic obstructive' OR 'pulmonary disorder, chronic obstructive' OR 'chronic obstructive lung disease')

#2 ('withdrawal, treatment' OR 'withholding treatment' OR 'treatment withdrawal') OR (de escalation) OR (step down therapy)

#3 ('administration, inhalation' OR 'aerosol therapy' OR 'dose inhalation' OR 'drug administration, inhalational' OR 'drug inhalation' OR 'inhalant administration' OR 'inhalant therapy' OR 'inhalant treatment' OR 'inhalation administration' OR 'inhalation drug administration' OR 'inhalation medication' OR 'inhalation therapy' OR 'inhalation treatment' OR 'inhalational administration' OR 'inhaled dosage' OR 'inhaled dose' OR 'inhaled drug' OR 'vapor therapy' OR 'vapour therapy' OR 'inhalational drug administration')

#4 ('glucocorticoid drug' OR 'glucocorticoid hormone' OR 'glucocorticoid steroid' OR 'glucocorticoids' OR 'glucocorticoids, synthetic' OR 'glucocorticoids, topical' OR 'glucocorticoidsteroid' OR 'glucocorticosteroid' OR 'glucocortoid' OR 'glycocorticoid' OR 'glycocorticosteroid' OR 'glucocorticoid') OR ('9 chloro 11beta, 17, 21 trihydroxy 16beta methylpregna 1, 4 diene 3, 20 dione' OR '9alpha chloro 16beta methylprednisolone' OR 'beclomethasone' OR 'prednisolone, 9alpha chloro 16beta methyl' OR 'pregna 1, 4 diene 11beta, 17, 21 triol 3, 20 dione, 9 chloro 16beta methyl' OR 'beclometasone') OR ('11 hydroxy 8 (2 hydroxyacetyl) 9, 13 dimethyl 6 propyl 5, 7 dioxapentacyclo [10.8.0.0 (2, 9) .0 (4, 8) .0 (13, 18)] icosa 14, 17 dien 16 one' OR '11beta, 16alpha, 17, 21 tetrahydroxypregn 1, 4 diene 3, 20 dione 16, 17 butyraldehyde cyclic acetal' OR '11beta, 16alpha, 17, 21 tetrahydroxypregna 1, 4 diene 3, 20 dione 16, 17 butyraldehyde cyclic acetal' OR '11beta, 16alpha, 17, 21 tetrahydroxypregna 1, 4 diene 3, 20 dione cyclic 16, 17 acetal with butyraldehyde' OR '16, 17 butylidenebis (oxy) 11, 21 dihydroxypregna 1, 4 diene 3, 20 dione' OR '16alpha, 17beta butylidenedioxy 11beta, 21 dihydroxypregna 1, 4 diene 3, 20 dione' OR '6b glycoloyl 5 hydroxy 4a, 6a dimethyl 8 propyl 4a, 4b, 5, 6, 6a, 6b, 9a, 10, 10a, 10b, 11, 12 dodecahydro 2h naphtho [2`, 1`:4, 5] indeno [1, 2 d] [1, 3] dioxol 2 one' OR 'acorspray' OR 'aerox' OR 'airbudenid' OR 'aircort' OR 'allercort' OR 'aq 001s' OR 'aq001s' OR 'aquacort' OR 'aurid' OR 'axelovert' OR 'b cort' OR 'bebe cream' OR 'benacort' OR 'benarhin' OR 'benodil' OR 'bidien' OR 'biosonide' OR 'bodinet' OR 'bodix' OR 'budapp' OR 'budased' OR 'budecol' OR 'budecort' OR 'budecort nasal' OR 'budecort novolizer' OR 'budecort nt' OR 'budefat' OR 'budeflam' OR 'budelin' OR 'budelin novolizer' OR 'budelite' OR 'budena' OR 'budenase aq' OR 'budenite' OR 'budeno' OR 'budenobronch' OR 'budenofalk' OR 'budenoside' OR 'budeprol' OR 'buderap bp' OR 'buderen' OR 'budes' OR 'budesoderm' OR 'budesolv' OR 'budeson' OR 'budeson 3' OR 'budesonal' OR 'budesonide easyhaler' OR 'budevin' OR 'budevin bp' OR 'budexan' OR 'budiair' OR 'budicort respules' OR 'budineb' OR 'budixon neb' OR 'budo-san' OR 'budon' OR 'budosan' OR 'budsocare' OR 'bunase' OR 'buparid' OR 'butacort' OR 'butacort aqueous' OR 'butekont' OR 'butolir' OR 'chf 1536' OR 'chf1536' OR 'clebudan' OR 'coramen' OR 'cortiment' OR 'cortiment mmx' OR 'cortimentmmx' OR 'cortivent' OR 'cycortide' OR 'd 9421c' OR 'd9421c' OR 'desona nasal' OR 'desonix' OR 'dexbudesonide' OR 'duasma' OR 'eltair' OR 'entocir' OR 'entocord' OR 'entocort' OR 'entocort ec' OR 'eohilia' OR 'eolan' OR 'esonide' OR 'fe 999315' OR 'fe999315' OR 'frenolyn' OR 'giona easyhaler' OR 'inflammide' OR 'inflanaze' OR 'intesticort' OR 'intesticortmono' OR 'intestifalk' OR 'jorveza' OR 'kesol' OR 'kinpeygo' OR 'larbex' OR 'lisobron' OR 'livicort' OR 'ludonase b' OR 'map 0010' OR 'map0010' OR 'micronyl' OR 'miflo' OR 'miflonid' OR 'miflonide' OR 'miflonide breezhaler' OR 'miflonide inhaler' OR 'miflonil' OR 'miflonil breezhaler' OR 'mikicort' OR 'nebbud' OR 'nebulin (drug)' OR 'nefecon' OR 'neo-rinactive' OR 'novopulmon' OR 'novopulmon novolizer' OR 'numark' OR 'obecirol' OR 'obusonid' OR 'olfex' OR 'olfex bucal' OR 'olfex bucal infantil' OR 'olfosonide' OR 'ortikos' OR 'pl 56' OR 'pl56' OR 'preferid' OR 'pregna 1, 4 diene 3, 20 dione 16, 17 butyraldehyde cyclic acetal, 11beta, 16alpha, 17, 21 tetrahydroxy' OR 'pt 008' OR 'pt008' OR 'pulmaxan' OR 'pulmicon susp for nebuliser' OR 'pulmicon susp for nebulizer' OR 'pulmicort' OR 'pulmicort flexhaler' OR 'pulmicort nasal' OR 'pulmicort nasal aqua' OR 'pulmicort nasal turbohaler' OR 'pulmicort respules' OR 'pulmicort turbohaler' OR 'pulmicort turbuhaler' OR 'pulmictan' OR 'pulmihal' OR 'pulmoliseflam' OR 'pulmotide' OR 'pulmovance' OR 'resata' OR 'respicort' OR 'rhinobros' OR 'rhinocort' OR 'rhinocort allergy' OR 'rhinocort alpha' OR 'rhinocort aqua' OR 'rhinocort aqueous' OR 'rhinocort hayfever' OR 'rhinocort turbohaler' OR 'rhinocort turbuhaler' OR 'rhinoside' OR 'ribujet' OR 'ribuspir' OR 'ribuvent' OR 'rinoster (drug)' OR 's 1320' OR 's1320' OR 'serbo' OR 'shp 621' OR 'shp621' OR 'spirocort' OR 'spirocort turbuhaler' OR 'tafen nasal' OR 'tak 721' OR 'tak721' OR 'talgan' OR 'tarpeyo' OR 'tinkair' OR 'uceris' OR 'velorium' OR 'vericort' OR 'vernoral' OR 'vinecort' OR 'weldinide' OR 'zefecort'

OR 'zentacort' OR 'zyolaif' OR 'budesonide') OR ('6, 9 difluoro 11 hydroxy 16 methyl 3 oxo 17 (1 oxopropoxy) androsta 1, 4 diene 17 carbothioic acid s (fluoromethyl) ester' OR '6alpha, 9 difluoro 17beta [[(fluoromethyl) sulfanyl] carbonyl] 11beta hydroxy 16 methyl 3 oxoandrosta 1, 4 dien 17alpha yl propanoate' OR '[6, 9 difluoro 17 (fluoromethylsulfanylcarbonyl) 11 hydroxy 10, 13, 16 trimethyl 3 oxo 6, 7, 8, 11, 12, 14, 15, 16 octahydrocyclopenta [a] phenanthren 17 yl] propanoate' OR 'ac 155' OR 'ac155' OR 'aerosona' OR 'apc 4000' OR 'apc4000' OR 'apt 1011' OR 'apt1011' OR 'armonair digihaler' OR 'armonair respiclick' OR 'arquist' OR 'asmatil' OR 'asmatil diskus' OR 'asmo-lavi' OR 'asmo-lavi diskus' OR 'atemur' OR 'atemur mite' OR 'axotide' OR 'beconase allergy 24 hour' OR 'brethal' OR 'brisovent' OR 'brisovent diskus' OR 'brisovent inalador' OR 'casoflune' OR 'cci 18781' OR 'cci18781' OR 'cortifil' OR 'cultivate' OR 'cutivat' OR 'cutivate' OR 'eds-flu' OR 'ep 104iar' OR 'ep104iar' OR 'eur 1100' OR 'eur1100' OR 'eustidil (fluticasone propionate)' OR 'fanipos' OR 'flixoderm' OR 'flixonase' OR 'flixonase 24 hour' OR 'flixonase allergia' OR 'flixonase allergy' OR 'flixonase aqua' OR 'flixonase ari' OR 'flixonase nasal spray' OR 'flixonase nasule' OR 'flixotaide' OR 'flixotaide diskus' OR 'flixotaide inalador' OR 'flixotaide nebules' OR 'flixotide' OR 'flixotide accuhaler' OR 'flixotide disk' OR 'flixotide disks' OR 'flixotide diskus' OR 'flixotide diskus junior' OR 'flixotide dysk' OR 'flixotide evohaler' OR 'flixotide forte' OR 'flixotide inhaler' OR 'flixotide junior' OR 'flixotide nebules' OR 'flixotide standard' OR 'flixovate' OR 'floebb inhaler' OR 'flonase' OR 'flonase allergy' OR 'flonase allergy relief' OR 'flovent' OR 'flovent diskus' OR 'flovent diskus 100' OR 'flovent diskus 250' OR 'flovent diskus 50' OR 'flovent hfa' OR 'flovent rotadisk' OR 'flugenix' OR 'fluinol' OR 'flunase' OR 'flunase aqueous' OR 'flunutrac' OR 'flusonal' OR 'flusonal accuhaler' OR 'fluspiral' OR 'flutaide' OR 'flutica-teva' OR 'fluticasone 17 propionate' OR 'fluticrem' OR 'flutide' OR 'flutide diskus' OR 'flutide evohaler' OR 'flutide nasal' OR 'flutinase' OR 'flutirin' OR 'flutivate' OR 'fluxonal' OR 'gr 18781' OR 'gr18781' OR 'inalacor' OR 'inalacor accuhaler' OR 'nasofan' OR 'nasofan aqueous' OR 'ncx 4251' OR 'ncx4251' OR 'opn 375' OR 'opn375' OR 'optinose' OR 'ot 503' OR 'ot503' OR 'otri allergie (fluticasone propionate)' OR 'otri-allergie (fluticasone propionate)' OR 'pavetod' OR 'pf 00241939' OR 'pf 04764793' OR 'pf 241939' OR 'pf 4764793' OR 'pf00241939' OR 'pf04764793' OR 'pf241939' OR 'pf4764793' OR 'pirinase hayfever relief' OR 'prutica' OR 'reviflut' OR 'reviflut axahaler' OR 'rinosone' OR 's (fluoromethyl) 6alpha, 9 difluoro 11beta, 17 dihydroxy 16alpha methyl 3 oxoandrosta 1, 4 diene 17beta carbothioate 17 propionate' OR 'sonera' OR 'trialona' OR 'trialona accuhaler' OR 'truflo' OR 'ubizol' OR 'xhance' OR 'zoflut' OR 'fluticasone propionate') OR ('alisade' OR 'allermist' OR 'arnuity' OR 'arnuity ellipta' OR 'avamys' OR 'breemista' OR 'ennhale' OR 'flonase sensimist' OR 'flonase sensimist allergy relief' OR 'fluticasone 17 furoate' OR 'fluticasone furoate gsk' OR 'furamist' OR 'furamyst' OR 'gsk 685698' OR 'gsk685698' OR 'gw 685698' OR 'gw 685698x' OR 'gw685698' OR 'gw685698x' OR 'veramyst' OR 'fluticasone furoate')

Final query: #1 AND #2 AND #3 AND #4

Supplementary S2. Definition of the outcomes of interest in the studies included in the meta-analysis.

**Study**	**Definition of acute exacerbation of COPD**
Magnussen, 2014[1]<br>Watz, 2016[2]	An increase or new onset of ≥ 2 lower respiratory symptoms related to COPD, with ≥ 1 symptom lasting ≥ 3 days requiring a change in treatment"<br>Lower respiratory symptoms include:<br>• Shortness of breath;<br>• Sputum production (volume);<br>• Sputum purulence;<br>• Cough;<br>• Wheezing;<br>• Chest tightness;<br>A change in treatment includes the following:<br>• Hospitalisation/ Treatment in Urgent Care Unit (severe exacerbation);<br>• Prescription of antibiotics and/ or systemic steroids (moderate exacerbation);<br>• Significant change of prescribed respiratory medication (i.e: theophyllines, long-acting beta-agonists, inhaled corticosteroids) (mild exacerbation);
Chapman, 2018[3]	Exacerbations, defined according to Anthonisen criteria[4], were categorized as mild (worsening of symptoms for >2 consecutive days and not treated with systemic corticosteroids and/or antibiotics), moderate (treated with systemic corticosteroids and/or antibiotics), or severe (requiring hospitalization [or an emergency room visit of 24 h] in addition to treatment with systemic corticosteroids and/or antibiotics).
Han, 2020[5]	COPD exacerbations are defined as follows:<br>• Mild: Worsening symptoms of COPD that are self-managed by the subject. Mild exacerbations are not associated with the use of corticosteroids or antibiotics;<br>• Moderate: Worsening symptoms of COPD that require treatment with oral/systemic corticosteroids and/or antibiotics;<br>• Severe: Worsening symptoms of COPD that require treatment with in-patient Hospitalization;
Vogelmaier, 2022[6]	N/A.
Magnussen, 2021[7]	An exacerbation was defined as: an unscheduled hospital admission or accident and emergency (A&E) attendance for COPD/respiratory condition or generic hospitalisation code* on the same day as a lower respiratory coded* consultation, course of oral steroids and/or antibiotics prescribed with lower respiratory consultation. More than one oral steroid course, A&E attendance, hospitalisation or prescription for antibiotics occurring within 2 weeks of each other were considered the result of the same exacerbation and were only be counted once. *coded on Optimum Patient Care Research Database (OPCRD).
Whittaker, 2022[8]	N/A for the purpose of our meta-analysis.
**Study**	**Definition of change from baseline in trough FEV_1**
Magnussen, 2014[1]<br>Watz, 2016[2]	Difference between FEV_1 measurement at week 0 and 52. Not clearly stated the "trough" FEV_1 definition.
Chapman, 2018[3]	Difference between FEV_1 measurement at week 0 and 26 (a mean of the two FEV_1 values measured at 23 h 15 min and 23 h 45 min after the morning dose on Day 181).
Han, 2020[5]	N/A.
Vogelmaier, 2022[6]	N/A.
Magnussen, 2021[7]	N/A
Whittaker, 2022[8]	N/A
**Study**	**Definition of change from baseline in FEV_1 recorded anytime**
Magnussen, 2014[1]<br>Watz, 2016[2]	N/A
Chapman, 2018[3]	N/A
Han, 2020[5]	N/A
Vogelmaier, 2022[6]	N/A
Magnussen, 2021[7]	Annualised change in FEV_1, where baseline FEV_1 was recorded anytime in the baseline year and outcome FEV_1 was recorded between 9 and 15 months post-index prescription date.
Whittaker, 2022[8]	Difference between FEV_1 recorded on Clinical Practice Research Datalink (CPRD) at least 6 months apart.
**Study**	**Definition of pneumonia**
Magnussen, 2014[1]<br>Watz, 2016[2]	A chest X-ray was performed for all patients with a suspected diagnosis of pneumonia;
Chapman, 2018[3]	Pneumonia was defined as an event characterized by increased respiratory symptoms (e.g. increased cough, dyspnea, wheezing, purulent sputum and fever) (i.e. body temperature greater than 38°C) or pleuritic chest pain or leukocytosis or other clinical signs consistent with pneumonia considered relevant in the opinion of the investigator. Radiographic imaging (chest X-ray or CT scan) was required to confirm the diagnosis. The diagnosis of COPD exacerbation will not preclude a diagnosis of pneumonia.
Han, 2020[5]	All suspected pneumonias will require confirmation as defined by the presence of new infiltrate(s) on chest x-ray and at least 2 of the following signs and symptoms:<br>• Increased cough;<br>• Increased sputum purulence (color) or production;<br>• Auscultatory findings of adventitious sounds (e,g, egophony, bronchial breath sounds, rales, etc.);<br>• Dyspnea or tachypnea;

	• Fever (oral temperature > 37.5 °C);<br>• Elevated WBC (>10,000/mm3 or >15% immature forms);<br>• Hypoxemia (HbO_2 saturation ≤88% or at least 2% lower than baseline value);
Vogelmaier, 2022[6]	Not specified.
Magnussen, 2021[7]	Pneumonia consultation and pneumonia coded on OPCRD.
Whittaker, 2022[8]	N/A for the purpose of our meta-analysis.
**Study**	**Definition of all-cause mortality**
Magnussen, 2014[1]<br>Watz, 2016[2]	Death from any cause.
Chapman, 2018[3]	
Han, 2020[5]	
Vogelmaier, 2022[6]	
Magnussen, 2021[7]	
Whittaker, 2022[8]	

COPD: Chronic obstructive pulmonary disease; N/A: not applicable, stated or reported; FEV_1: forced espiratory Volume in the first second; X-ray: radiography; CT: computed tomography; WBC: white blood count; HbO_2: oxyhemoglobin;

Supplementary S3. List of studies excluded at the full-text screening stage with the reason for exclusion.

Title of Study	Reason for exclusion
A Cluster Randomised Trial of Medication Review and Withdrawal of Inappropriate Inhaled Corticosteroid Treatment in Chronic Obstructive Pulmonary Disease, NCT03489746	Study withdrawn.
Miravitlles M, Verhamme K, Calverley P, et al. A Pooled Analysis of Mortality in Patients with COPD Receiving Dual Bronchodilation with and without Additional Inhaled Corticosteroid. INTERNATIONAL JOURNAL OF CHRONIC OBSTRUCTIVE PULMONARY DISEASE 2022; 17: 545–58	Different intervention.
Miravitlles M, Verhamme K, Calverley P, et al. A Pooled Analysis of Mortality in Patients with COPD Receiving Dual Bronchodilation with and without Additional Inhaled Corticosteroid Am J Respir Crit Care Med 2021;203:A2251	Conference abstract.
Vafai-Tabrizi F, Schwab U, Brecht S, Funk G-C. Adjustments to maintenance therapy and the reasoning behind them among COPD outpatients in Austria: the STEP study. ERJ Open Res 2024; 10. DOI:10.1183/23120541.00615-2023.	Different intervention.
Lee S, Lee J, Yoon H, et al. Change in inhaled corticosteroid treatment and COPD exacerbations: an analysis of real-world data from the KOLD/KOCOSS cohorts. RESPIRATORY RESEARCH 2019; 20. DOI:10.1186/s12931-019-1029-7	Cannot extract data. No response from corresponding author.
Garcia V, Vallejo-Aparicio L, Ismaila A, et al. Clinical and Economic Impact of Long-Term Inhaled Corticosteroid Withdrawal in Patients with Chronic Obstructive Pulmonary Disease Treated with Triple Therapy in Spain. INTERNATIONAL JOURNAL OF CHRONIC OBSTRUCTIVE PULMONARY DISEASE 2022; 17: 2161–74.	Different intervention.
Suissa S, Dell'Aniello S, Ernst P. Discontinuation of Inhaled Corticosteroids from Triple Therapy in COPD: Effects on Major Outcomes in Real World Clinical Practice. COPD J Chronic Obstructive Pulm Dis 2022; 19: 133–41.	Different intervention.
Reilev M, Kristensen K, Sondergaard J, Henriksen D, Thompson W, Pottegård A. Discontinuation of therapy among COPD patients who experience an improvement in exacerbation status. EUROPEAN JOURNAL OF CLINICAL PHARMACOLOGY 2019; 75: 1025–32	Different intervention.
Buhl R, Criée C-P, Kardos P, et al. Dual bronchodilation vs triple therapy in the "real-life" COPD DACCORD study. Int J COPD 2018; 13: 2557–68.	Different intervention/sub-group analysis.
Worth H, Buhl R, Criée C-P, Kardos P, Lossi NS, Vogelmeier CF. GOLD 2017 treatment pathways in 'real life': An analysis of the DACCORD observational study. Respir Med 2017; 131: 77–84.	Subgroup analysis.
Bloom CI, Douglas I, Usmani OS, Quint JK. Inhaled Corticosteroid Treatment Regimens and Health Outcomes in a UK COPD Population Study. Int J Chron Obstruct Pulmon Dis 2020; 15: 701–10.	Different intervention.
Magnussen H, Tetzlaff K, Bateman E, et al. Lung function changes over time following withdrawal of inhaled corticosteroids in patients with severe COPD. The european respiratory journal 2016; 47: 651-654.	Letter to the editor.
Wouters E, Magnussen H, Rodriguez-Roisin R, Tetzlaff K, Bell S, Calverley P. Lung-function profile before and after the first moderate to severe exacerbation during the wisdom study. American Journal of Respiratory and Critical Care Medicine 2016;193:A5179	Conference abstract.
Vogelmeier C, Worth H, Buhl R, et al. 'Real-life' inhaled corticosteroid withdrawal in COPD: a subgroup analysis of DACCORD. INTERNATIONAL JOURNAL OF CHRONIC OBSTRUCTIVE PULMONARY DISEASE 2017; 12: 487–94.	Different intervention.
Nielsen AO, Hilberg O, Jensen JUS, et al. Withdrawal of inhaled corticosteroids in patients with COPD – A prospective observational study. Int J COPD 2021; 16: 807–15.	Different intervention.
Kim SA, Lee J-H, Kim E-K, et al. Outcome of Inhaler Withdrawal in Patients Receiving Triple Therapy for COPD. Tuberc Respir Dis (Seoul) 2016; 79: 22–30.	Different intervention.
Kostikas K, Hurst J, Chapman K, et al. Persistent blood eosinophilia and copd exacerbation risk after ICS withdrawal from triple therapy in the sunset study. Respirology (Carlton, Vic) 2019; 24: 51.	Conference abstract.
Watz H, Magnussen H, Rodriguez-Roisin R, et al. Subgroup analyses of lungfunction change from the WISDOM study. Chest 2015; 148. DOI:10.1378/chest.2277533.	Conference abstract.

Supplementary S4. Forest plot of additional data from analysed outcomes.

Supplementary S4.1. Forest plot of subset analysis of the randomized controlled trials comparing time to first moderate or severe acute exacerbations in de-escalation from triple therapy with ICS withdrawal group vs triple therapy group continuation according to absolute count of eosinophils.

Study or Subgroup	log[HR]	SE	ICS withdrawal Total	ICS/LABA/LAMA Total	Weight	Hazard ratio IV, Random, 95% CI
**1.9.1 Eos<300 cell/μL**						
Chapman 2018	-0.051293	0.155951	401	406	25.6%	0.95 [0.70 , 1.29]
Watz 2016	0.076035	0.075643	902	889	37.9%	1.08 [0.93 , 1.25]
**Subtotal**			**1303**	**1295**	**63.5%**	**1.05 [0.92 , 1.20]**
Test for overall effect: Z = 0.76 (P = 0.45)						
Heterogeneity: Tau² = 0.00; Chi² = 0.54, df = 1 (P = 0.46); I² = 0%						
**1.9.2 Eos≥300 cell/μL**						
Chapman 2018	0.587787	0.308181	125	119	11.4%	1.80 [0.98 , 3.29]
Watz 2016	0.426574	0.159177	222	226	25.1%	1.53 [1.12 , 2.09]
**Subtotal**			**347**	**345**	**36.5%**	**1.58 [1.20 , 2.09]**
Test for overall effect: Z = 3.26 (P = 0.001)						
Heterogeneity: Tau² = 0.00; Chi² = 0.22, df = 1 (P = 0.64); I² = 0%						
**Total**			**1650**	**1640**	**100.0%**	**1.21 [0.95 , 1.53]**

Test for overall effect: Z = 1.57 (P = 0.12)
Test for subgroup differences: Chi² = 6.78, df = 1 (P = 0.009), I² = 85.3%
Heterogeneity: Tau² = 0.03; Chi² = 7.54, df = 3 (P = 0.06); I² = 60%

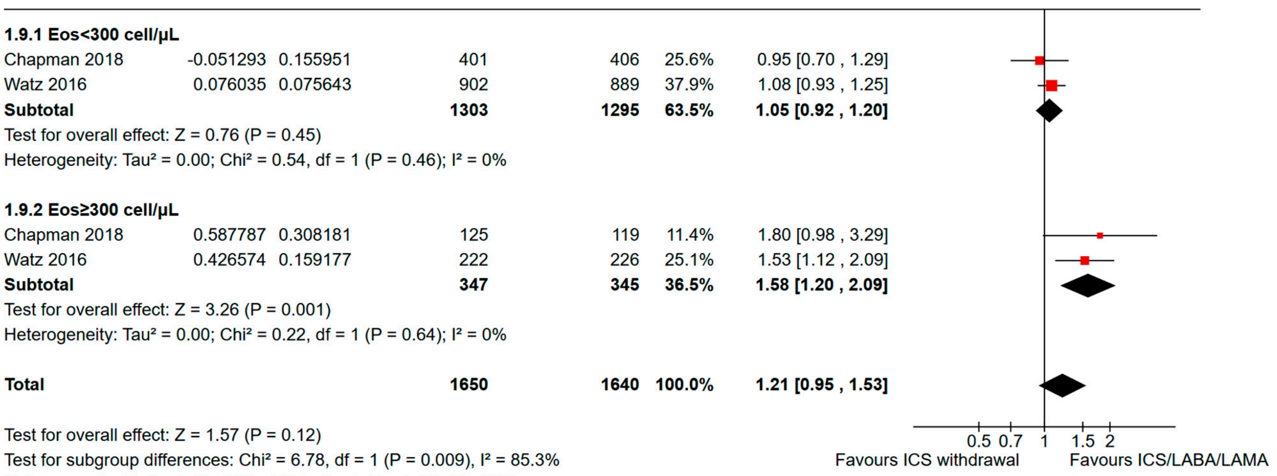

Supplementary S4.2. Forest plot of meta-analysis comparing event rate of moderate or severe acute exacerbations in de-escalation from triple therapy with ICS withdrawal group vs triple therapy group continuation according to percentage of eosinophil counts.

Study or Subgroup	log[Rate ratio]	SE	ICS withdrawal Total	ICS/LABA/LAMA Total	Weight	Rate ratio IV, Random, 95% CI
**1.3.1 Eos<2%**						
Chapman 2018	-0.150823	0.285417	131	113	4.8%	0.86 [0.49 , 1.50]
Watz 2016	0.019803	0.104459	528	511	35.8%	1.02 [0.83 , 1.25]
**Subtotal**			**659**	**624**	**40.6%**	**1.00 [0.82 , 1.21]**
Test for overall effect: Z = 0.00 (P = 1.00)						
Heterogeneity: Tau² = 0.00; Chi² = 0.32, df = 1 (P = 0.57); I² = 0%						
**1.3.2 Eos≥2%**						
Chapman 2018	0.198851	0.154629	395	412	16.4%	1.22 [0.90 , 1.65]
Watz 2016	0.202124	0.09534	596	604	43.0%	1.22 [1.02 , 1.48]
**Subtotal**			**991**	**1016**	**59.4%**	**1.22 [1.04 , 1.43]**
Test for overall effect: Z = 2.48 (P = 0.01)						
Heterogeneity: Tau² = 0.00; Chi² = 0.00, df = 1 (P = 0.99); I² = 0%						
**Total**			**1650**	**1640**	**100.0%**	**1.13 [1.00 , 1.27]**

Test for overall effect: Z = 1.91 (P = 0.06)
Test for subgroup differences: Chi² = 2.51, df = 1 (P = 0.11), I² = 60.1%
Heterogeneity: Tau² = 0.00; Chi² = 2.82, df = 3 (P = 0.42); I² = 0%

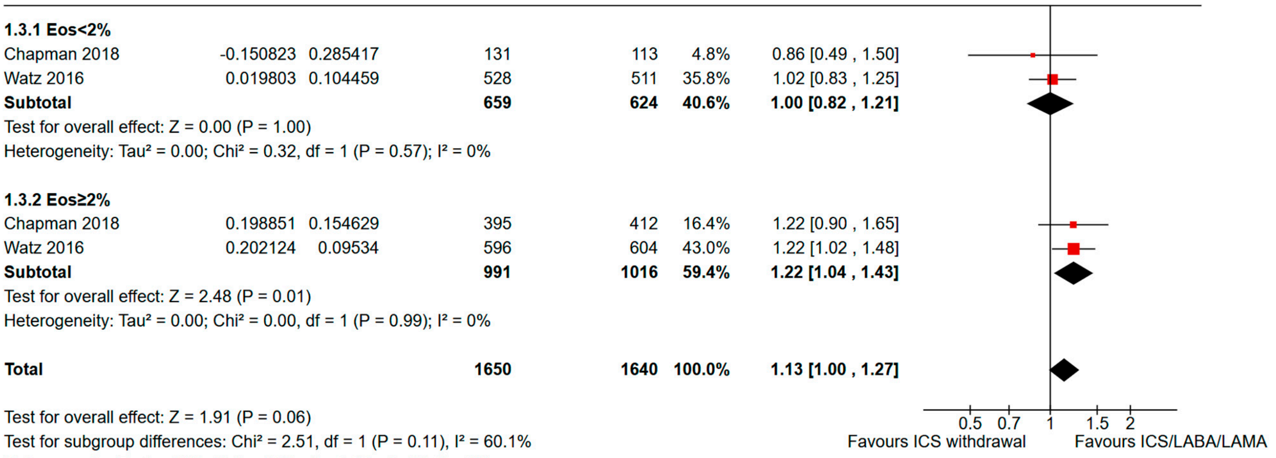

Supplementary S4.3. Forest plot of meta-analysis comparing change from baseline trough FEV_1 in de-escalation from triple therapy with ICS withdrawal group vs triple therapy continuation group according to percentage of eosinophil counts.

Study or Subgroup	MD	SE	Weight	Mean difference IV, Random, 95% CI
**2.2.1 Eos<2%**				
Chapman 2018	41	28.171633	18.4%	41.00 [-14.22 , 96.22]
Watz 2016	-43	20.249975	25.0%	-43.00 [-82.69 , -3.31]
**Subtotal (95% CI)**			**43.4%**	**-3.28 [-85.48 , 78.91]**
Heterogeneity: Tau² = 2926.15; Chi² = 5.86, df = 1 (P = 0.02); I² = 83%				
Test for overall effect: Z = 0.08 (P = 0.94)				
**2.2.2 Eos≥2%**				
Chapman 2018	-45	15.53705	29.7%	-45.00 [-75.45 , -14.55]
Watz 2016	-43	18.30294	26.9%	-43.00 [-78.87 , -7.13]
**Subtotal (95% CI)**			**56.6%**	**-44.16 [-67.38 , -20.95]**
Heterogeneity: Tau² = 0.00; Chi² = 0.01, df = 1 (P = 0.93); I² = 0%				
Test for overall effect: Z = 3.73 (P = 0.0002)				
**Total (95% CI)**			**100.0%**	**-28.17 [-60.09 , 3.76]**
Heterogeneity: Tau² = 651.13; Chi² = 8.03, df = 3 (P = 0.05); I² = 63%				
Test for overall effect: Z = 1.73 (P = 0.08)				
Test for subgroup differences: Chi² = 0.88, df = 1 (P = 0.35), I² = 0%				

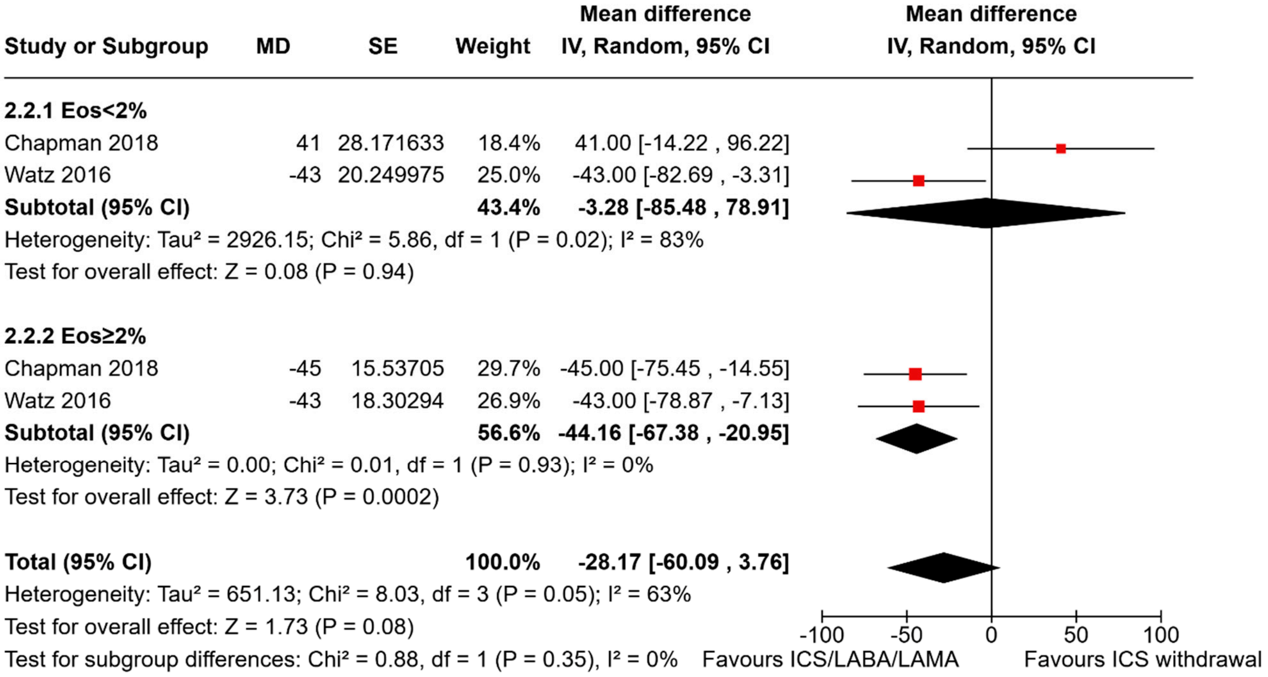

Supplementary S4.4. Forest plot of meta-analysis comparing change from baseline FEV_1 in de-escalation from triple therapy with ICS withdrawal group vs triple therapy continuation group with Cohort 1 of Whittaker et al[8]**.**

Study or Subgroup	ICS/LABA/LAMA Mean [mL]	SD [mL]	Total	ICS withdrawal Mean [mL]	SD [mL]	Total	Weight	Mean difference IV, Random, 95% CI [mL]
Magnussen 2021	-18.8	253.2	4184	-48.8	226.4	1046	49.0%	30.00 [14.28 , 45.72]
Whittaker 2022	-13.9	128.270965	5470	-7.4	136.378243	538	51.0%	-6.50 [-18.51 , 5.51]
**Total (95% CI)**			**9654**			**1584**	**100.0%**	**11.38 [-24.38 , 47.15]**
Heterogeneity: Tau² = 615.17; Chi² = 13.07, df = 1 (P = 0.0003); I² = 92%								
Test for overall effect: Z = 0.62 (P = 0.53)								
Test for subgroup differences: Not applicable								

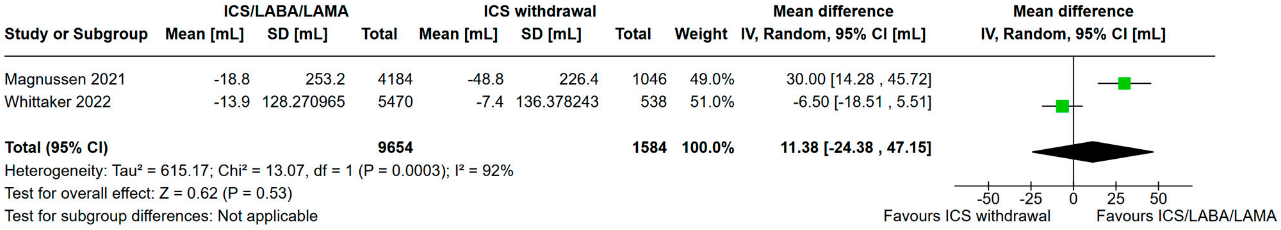

Supplementary S5. Jackknife sensitivity analysis.

Supplementary S5.1. Moderate or severe acute exacerbations (Hazard Ratio).

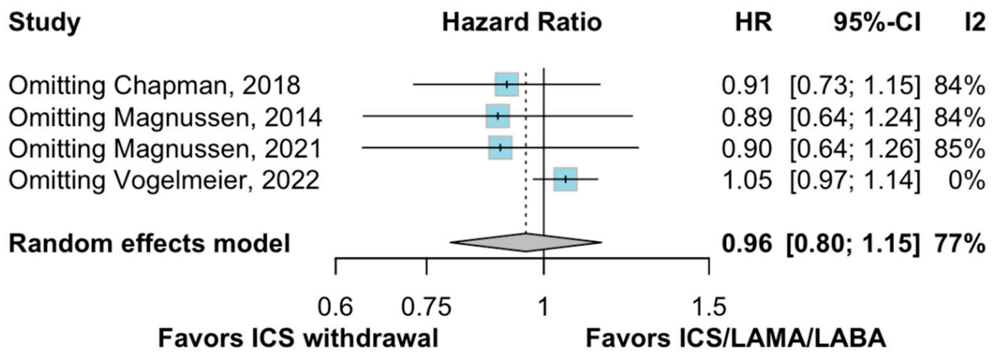

Supplementary S5.2. Moderate or severe acute exacerbations (Rate Ratio).

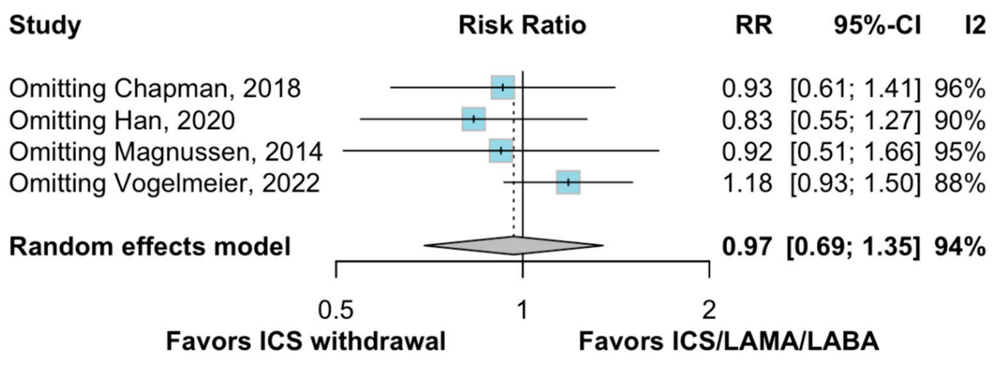

Supplementary S5.3. Moderate or severe acute exacerbation (Eos<300 cell/µL - Hazard Ratio).

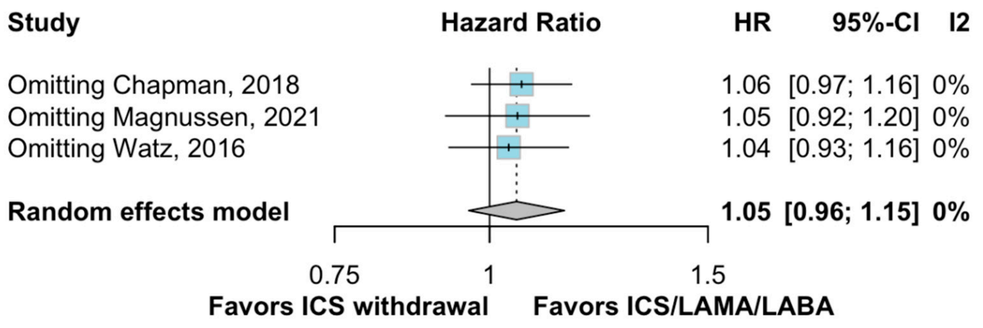

Supplementary S5.4. Moderate or severe acute exacerbation (Eos≥300 cell/µL - Hazard Ratio).

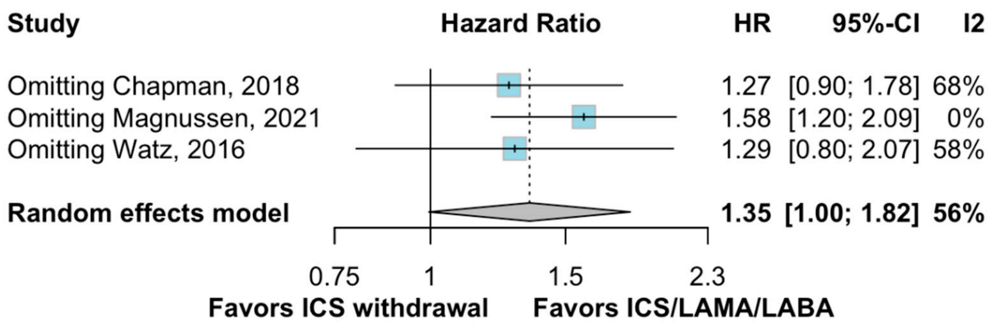

Supplementary S5.5. Odds ratio of occurrence of pneumonia.

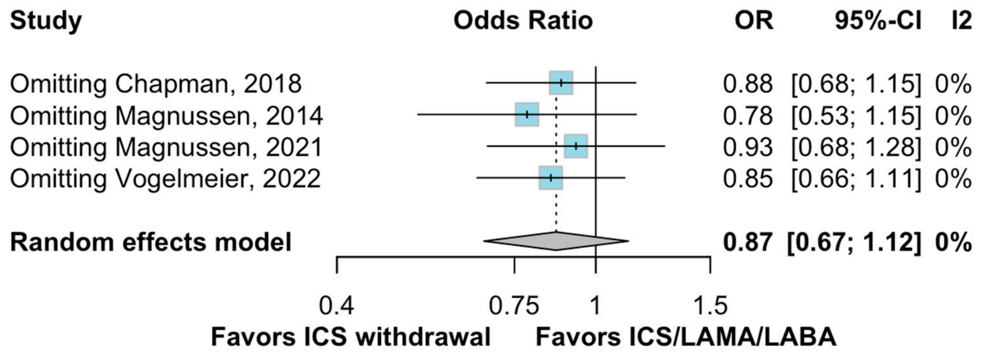

Supplementary S5.6. Odds ratio of occurrence of all-cause mortality.

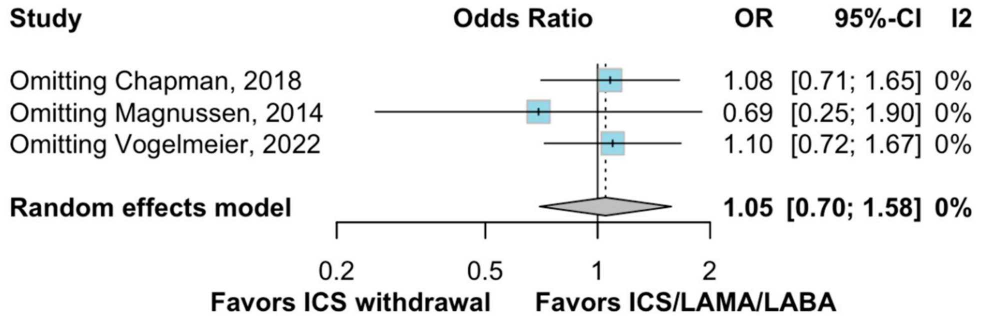

Supplementary S6. Risk of bias assessment.

Supplementary S6.1 Risk of bias 2 (RoB2)[9].

Study	Bias from randomization process	Bias due to deviations from intended interventions	Bias due to missing outcome data	Bias in measurement of the outcomes	Bias in selection of the reported result	Overall risk of bias
Magnussen, 2014[1]<br><br>Watz, 2016[2]	Low	Low	Low	Some concerns	Low	Some concerns
Chapman, 2018[3]	Low	Low	Low	Low	Low	Low
Han, 2020[5]	Low	Low	Low	Low	Low	Low

Supplementary S6.2 Risk of bias summary for non-randomized studies (ROBINS-I)[10].

Study	Bias due to confounding	Bias in selection of participants	Bias in classification of interventions	Bias due to deviations from intended interventions	Bias due to missing data	Bias in measurement of outcomes	Bias in selection of the reported result	Overall risk of bias judgement
Magnussen, 2021[7]	Moderate	Low	Moderate	Moderate	Moderate	Serious	Low	Serious
Vogelmaier, 2022[6]	Moderate	Low	Moderate	Moderate	Moderate	Serious	Low	Serious
Whittaker, 2022[8]	Moderate	Low	Moderate	Low	Moderate	Serious	Low	Serious

Appendix references

1. Magnussen, H.; Disse, B.; Rodriguez-Roisin, R.; Kirsten, A.; Watz, H.; Tetzlaff, K.; Towse, L.; Finnigan, H.; Dahl, R.; Decramer, M.; et al. Withdrawal of Inhaled Glucocorticoids and Exacerbations of COPD. *NEW ENGLAND JOURNAL OF MEDICINE* **2014**, *371*, 1285–1294, doi:10.1056/NEJMoa1407154.
2. Watz, H.; Tetzlaff, K.; Wouters, E.; Kirsten, A.; Magnussen, H.; Rodriguez-Roisin, R.; Vogelmeier, C.; Fabbri, L.; Chanez, P.; Dahl, R.; et al. Blood Eosinophil Count and Exacerbations in Severe Chronic Obstructive Pulmonary Disease after Withdrawal of Inhaled Corticosteroids: A Post-Hoc Analysis of the WISDOM Trial. *LANCET RESPIRATORY MEDICINE* **2016**, *4*, 390–398, doi:10.1016/S2213-2600(16)00100-4.
3. Chapman, K.R.; Hurst, J.R.; Frent, S.-M.; Larbig, M.; Fogel, R.; Guerin, T.; Banerji, D.; Patalano, F.; Goyal, P.; Pfister, P.; et al. Long-Term Triple Therapy De-Escalation to Indacaterol/Glycopyrronium in Patients with Chronic Obstructive Pulmonary Disease (SUNSET): A Randomized, Double-Blind, Triple-Dummy Clinical Trial. *Am J Respir Crit Care Med* **2018**, *198*, 329–339, doi:10.1164/rccm.201803-0405OC.
4. Anthonisen, N.R.; Manfreda, J.; Warren, C.P.; Hershfield, E.S.; Harding, G.K.; Nelson, N.A. Antibiotic Therapy in Exacerbations of Chronic Obstructive Pulmonary Disease. *Ann Intern Med* **1987**, *106*, 196–204, doi:10.7326/0003-4819-106-2-196.
5. Han, M.; Criner, G.; Dransfield, M.; Halpin, D.; Jones, C.; Kilbride, S.; Lange, P.; Lettis, S.; Lipson, D.; Lomas, D.; et al. The Effect of Inhaled Corticosteroid Withdrawal and Baseline Inhaled Treatment on Exacerbations in the IMPACT Study A Randomized, Double-Blind, Multicenter Clinical Trial. *AMERICAN JOURNAL OF RESPIRATORY AND CRITICAL CARE MEDICINE* **2020**, *202*, 1237–1243, doi:10.1164/rccm.201912-2478OC.
6. Vogelmeier, C.F.; Worth, H.; Buhl, R.; Criée, C.-P.; Gückel, E.; Kardos, P. Impact of Switching from Triple Therapy to Dual Bronchodilation in COPD: The DACCORD ‘Real World’ Study. *Respir. Res.* **2022**, *23*, doi:10.1186/s12931-022-02037-2.
7. Magnussen, H.; Lucas, S.; Lapperre, T.; Quint, J.; Dandurand, R.; Roche, N.; Papi, A.; Price, D.; Miravitlles, M.; REG Withdrawal of Inhaled Corticosteroids versus Continuation of Triple Therapy in Patients with COPD in Real Life: Observational Comparative Effectiveness Study. *RESPIRATORY RESEARCH* **2021**, *22*, doi:10.1186/s12931-021-01615-0.
8. Whittaker, H.; Wing, K.; Douglas, I.; Kiddle, S.; Quint, J. Inhaled Corticosteroid Withdrawal and Change in Lung Function in Primary Care Patients with Chronic Obstructive Pulmonary Disease in England. *ANNALS OF THE AMERICAN THORACIC SOCIETY* **2022**, *19*, 1834–1841, doi:10.1513/AnnalsATS.202111-1238OC.

9. Sterne, J.A.C.; Savović, J.; Page, M.J.; Elbers, R.G.; Blencowe, N.S.; Boutron, I.; Cates, C.J.; Cheng, H.-Y.; Corbett, M.S.; Eldridge, S.M.; et al. RoB 2: A Revised Tool for Assessing Risk of Bias in Randomised Trials. *BMJ* **2019**, *366*, l4898, doi:10.1136/bmj.l4898.
10. Sterne, J.A.; Hernán, M.A.; Reeves, B.C.; Savović, J.; Berkman, N.D.; Viswanathan, M.; Henry, D.; Altman, D.G.; Ansari, M.T.; Boutron, I.; et al. ROBINS-I: A Tool for Assessing Risk of Bias in Non-Randomised Studies of Interventions. *BMJ* **2016**, *355*, i4919, doi:10.1136/bmj.i4919.
